# FcRn Expression in Endometrial Cancer and Its Association with Clinicopathologic Features

**DOI:** 10.3390/diagnostics13243660

**Published:** 2023-12-14

**Authors:** Dae Hyun Song, Juseok Yang, Cho Hee Kim, Min Hye Kim, Jae Yoon Jo, Jong Chul Baek

**Affiliations:** 1 Department of Pathology, Gyeongsang National University School of Medicine, Gyeongsang National University Changwon Hospital, 11, Samjeongja-ro, Seongsan-gu, Changwon-si 51472, Republic of Korea; golgy@hanmail.net (D.H.S.); joymine86@naver.com (M.H.K.); 2 Department of Pathology, Gyeongsang National University Hospital, Jinju 52727, Republic of Korea; 3 Institute of Medical Science, Gyeongsang National University, Jinju 52727, Republic of Korea; yangandshin@gmail.com (J.Y.); chohing9@gmail.com (C.H.K.); poodeeng@naver.com (J.Y.J.); 4 Department of Obstetrics and Gynecology, Gyeongsang National University Changwon Hospital, 11, Samjeongja-ro, Seongsan-gu, Changwon-si 51472, Republic of Korea; 5 Department of Obstetrics and Gynecology, Gyeongsang National University School of Medicine, Jinju 52727, Republic of Korea; 6 Department of Obstetrics and Gynecology, Gyeongsang National University Hospital, Jinju 52727, Republic of Korea

**Keywords:** endometrial cancer, neonatal Fc receptor, Ishikawa cell, prognostic factors

## Abstract

Background: Endometrial cancer (EC) has robust molecular diagnostic evidence that correlates well with prognosis. In various types of cancers, FcRn has been identified as an early marker for prognosis. This study aims to assess FcRn expression and its association with clinicopathological features in endometrial cancer. Materials and Methods: We employed a tissue microarray (TMA) from a retrospective cohort of 41 patients diagnosed with endometrioid endometrial cancer post hysterectomy between January 2002 and December 2009 at Gyeongsang National University Hospital. Relevant clinical data collection for the cohort involved reviewing patients’ electronic medical charts. FcRn expression in microarrays of patient EC tissue was examined in conjunction with clinicopathologic data. Experiments, including siRNA knock-down, PCR mRNA semiquantification, Western blot, and confluence change tests, were conducted on the Ishikawa cell line. Results: The overall FcRn expression rate in EC patients was 41.8%. FIGO stage showed a statistically significant relationship with FcRn expression, while age, lymphovascular invasion, myometrial invasion, and tumor size had no effect. In endometrioid cancer cells of FIGO stage IA, FcRn was less frequently expressed than in other high-staged EC patients (*p* = 0.021). In experiments on the Ishikawa cell line, the siRNA knock-down group exhibited quantitatively lower FCGRT mRNA expression and lower FcRn protein signal compared to the scrambled RNA control group. The change in confluence over time measured at three hotspots did not show a significant difference between groups. Conclusions: To the best of our knowledge, this study represents the initial assessment of FcRn expression in endometrioid EC samples. FcRn expression was significantly associated with the FIGO stage. Ishikawa cell line proliferation did not significantly change in response to decreased FcRn expression. Further studies are needed to elucidate FcRn expression in EC as a potential molecular parameter.

## 1. Introduction

Endometrial cancer (EC) is the most common gynecological malignancy, and ranks sixth among the most frequently diagnosed female cancers [1]. Over the past three decades, the global incidence of EC has markedly increased, while overall cancer-related mortality has consistently declined. These trends hinge largely on early cancer diagnosis, socioeconomic factors, racial disparities, and geographic characteristics, all of which serve as pivotal prognostic indicators [1,2]. The EC staging system encompasses histological subtypes and molecular phenotypes. Following the genomic approach introduced by The Cancer Genome Atlas (TCGA) in 2013 [3], molecular classification has garnered significant attention. Molecular analysis has propelled advancements in risk stratification for recurrence and personalized treatment, subsequently improving survival rates [4,5]. Following TCGA’s impactful molecular diagnosis classification in EC, the Proactive Molecular Risk Classifier for Endometrial Cancer (ProMisE) introduced a straightforward and clinically applicable molecular classification system in 2015. It incorporates p53, MMR proteins, and POLE exonuclease domain mutant hotspot sequences [6]. Current applicable biomarkers in EC include estrogen and progesterone receptors, the PI3K/AKT/mTOR pathway, and Programmed Death Ligand-1 (PD-L1). Therapeutic agents based on these molecular subtypes are now available [7]. The integration of molecular parameters has significantly advanced risk stratification for recurrence and personalized treatment in EC. The neonatal Fc receptor (FcRn) was initially discovered in the small intestine of neonatal rats [8]. FcRn facilitates endogenous molecular transportation [9,10], and is expressed in various human tissues, including the intestines, mammary glands, lungs, and placenta [11,12]. FcRn’s promising features in cancer treatment and molecular diagnosis are rooted in its abilities of albumin recruitment [13,14] and the transcytosis of immunoglobulins, extending the half-life of cancer drugs and antibody-based therapeutics [15,16]. Moreover, it plays a crucial role in antitumor immunity through the antigen-processing pathway [17]. Although FcRn has garnered robust scientific attention [18], its expression in cancer cells and feasibility as a prognostic factor have only been studied in limited types of malignancies, such as non-small cell lung cancer and rectal cancer [19,20].

In this study, FcRn expression in tissue microarray (TMA) samples from patients with EC was evaluated, and its association with clinicopathologic features was analyzed. Ishikawa cell line experiments were also conducted on FcRn and its gene expression.

## 2. Material and Methods

### 2.1. Patients

We utilized a TMA from a retrospective cohort of patients diagnosed with endometrioid EC post hysterectomy between January 2002 and December 2009 at Gyeongsang National University Hospital (GNUH), Jinju, Republic of Korea. Relevant clinical data for the cohort were obtained through the review of patients’ electronic medical charts. A total of 41 patients were enrolled, and their clinicopathologic characteristics, including age at diagnosis, menopausal status, tumor grade, myometrial invasion, and lymphovascular space invasion (LVSI), were collected. EC staging was determined based on the 2009 version of FIGO staging, as the latest version reported in 2023 was not applicable to our patient treated during 2002–2009 [7]. Molecular classification testing (POLEmut, MMRd, NSMP, p53abn) was not widely used in South Korea during this period. Pathologic diagnosis confirmation involved two pathologists, with tumor staging, determination of histologic type, and grade based on the Seventh Edition of the American Joint Committee on Cancer [21] and the Fourth Edition of the World Health Organization classification [22], respectively. This study received approval from the Institutional Review Board of Gyeongsang National University Hospital, and informed consent was obtained from patients (GNUH-2022-03-009).

### 2.2. TMA and Immunohistochemical Analysis

We selected sections of specimens showing the most typical pathological figures of regions among hematoxylin and eosin-stained endometrioid EC specimens. Following fixation of the surgical specimens in buffered neutral formalin (20%) overnight, the samples were embedded in paraffin blocks. A single 3 mm tissue core, representing prominent intratumoral regions, was selected and transplanted into a recipient TMA block. Immunohistochemistry for FcRn expression was conducted using 4 μm thick sections of the TMA blocks. The samples were stained with a monoclonal antibody for FcRn (dilution 1:50; sc-271745; Santa Cruz Biotechnology Inc., Dallas, TX, USA) utilizing a fully automated immunohistochemical staining machine (Benchmark Ultra, Ventana Medical Systems Inc., Tucson, AZ, USA). The result of FcRn staining was deemed positive by comparing the cytoplasmic staining status of cancer cells to adjacent stroma. In this study, the expression level of tumor cells was measured using stromal tissue around the tumor as an internal control. The degree of FcRn tumor cell expression was defined as positive if it was higher than that of the surrounding stromal tissue, and negative if it was lower. To enhance the reproducibility of the staining results, two pathologists confirmed the outcomes in a blinded manner.

### 2.3. Ishikawa Cell Line Experiments

#### 2.3.1. Cell Culture

Dr. M. Han (Busan, University of Dong-A) provided the human EC cell line Ishikawa. Ishikawa cells were cultured in Dulbecco’s modified Eagle’s medium (Gibco, CA, USA, 11995-065) supplemented with 10% fetal bovine serum (FBS Opti-Gold, GenDEPOT, Dawin bio, Hanam, Republic of Korea, F0900-050) and 1% penicillin–streptomycin (Corning, NY, USA, #30-002-CI) at 37 °C in 5% CO_2_.

#### 2.3.2. Knock-Down of FcRn

Ishikawa cells were cultured to 70–80% confluence in 60 mm dishes. The cells assigned to the knock-down group were transfected using Lipofectamine 3000 (Invitrogen, CA, USA, #L3000015) with human FCGRT siRNA (siFCGRT, Bioneer, Daejeon, Republic of Korea, #2217-1), while cells in the negative control group were transfected with scrambled siRNA (Bioneer, #SN-1002) at a final concentration of 25 nM. After a 24 h incubation, cells were retransfected using the same protocol. The cells were then incubated for 72 h before harvesting.

#### 2.3.3. Semiquantitative PCR Analysis

Total RNA was extracted using TRIzol reagent (Qiagen, Germantown, MD, USA). Quantification of total RNA was conducted using a QIAxpert (Qiagen), and 1 μg of total RNA was reverse transcribed to complementary DNA using the Maxime RT PreMix Kit (iNtRON, Maryland, USA, #25081). An equal amount of synthesized complementary DNA was utilized for semiquantitative PCR using the Maxime PCR PreMix kit (iNtRON, #25025). The FCGRT primers (Bioneer, #P219030) were obtained from Bioneer (Daejeon, Republic of Korea). The GAPDH primer sequences were as follows: forward, 5′-GTC CAC CAC CCT GTT GCT GTA G-3′; reverse, 5′-CAA GGT CAT CCA TGA CAA CTT TG-3′.

#### 2.3.4. Western Blot Analysis

Proteins were extracted using RIPA lysis buffer (Thermo Fisher Scientific, Waltham, MA, USA, #89900) with a protease inhibitor cocktail (Thermo Fisher Scientific, #78430). The total protein concentration of each cell lysate was measured with the BCA method using bovine serum albumin as a standard. Equal amounts of protein lysates (45 μg) were loaded on a denaturing polyacrylamide gel and then transferred to a nitrocellulose membrane. The primary antibodies used for immune-blot were FcRn (santacruz, #sc-271745) and GAPDH (abcam, #ab8245), followed by horseradish peroxidase-conjugated secondary antibodies, and developed by enhanced chemiluminescence reaction (Thermo Fisher Scientific, #32109). Digital chemiluminescence images were taken by automatic molecular imaging system (Fusion Solo, Vilber Inc., Eberhardzell, Germany).

#### 2.3.5. Cell Proliferation

Ishikawa cells were cultured to 70–80% confluence in 60 mm dishes. Following the knock-down of FcRn, cells underwent a 24 h starvation period, and cell proliferation was monitored for 48 h. Images were captured at two locations in each sample. Out of a total of six results, those with insufficient or excessive cell counts on either side were excluded. Cell proliferation dynamics were recorded using JuLITM Br (NanoEntek, Seoul, Republic of Korea).

### 2.4. Statistical Analysis

Correlation analyses of categorical variables utilized the chi-square test and Fisher’s exact test to determine the association between FcRn expression in EC samples and clinicopathological characteristics of the cohort. Statistical significance was defined at *p* < 0.05. Statistical analysis was conducted using SPSS (ver. 24.0; IBM Corp., Armonk, NY, USA) and R software (ver. 4.21; R Project for Statistical Computing, Vienna, Austria).

## 3. Results

### 3.1. Patient Characteristics

The clinicopathologic characteristics of patients are summarized in Table 1. The mean age at diagnosis was 51.0 years, with almost half of the patients in menopause. All cases were histologically confirmed as endometrioid EC, with the majority (38/41, 92.7%) having a low tumor grade (equivalent to grade 1 and 2 in a three-tiered system). According to the 2009 FIGO staging, 34 cases were allocated to stage I, with 26 (63.4%) classified as IA and 8 (19.5%) as IB. Stage IIA accounted for 4.9%, with two patients, and IIIA and IIIC each comprised 4.9% and 7.3%, with two and three patients, respectively. Tumor size analysis revealed that 19.5% had sizes less than 2 cm, 31.7% ranged from 2 to 4 cm, and 48.8% were 4 cm or larger. LVSI was confirmed in 12.2% of patients. Myometrial invasion was identified in less than half of the myometrium in 56.1% of patients, while more than half of the myometrial thickness showed invasion in 43.9%. FcRn expression was identified in tumor cells of 41.5% of all patients (17 out of 41).

### 3.2. Correlation of FcRn Expression with Clinicopathological Features

**Figure 1:**
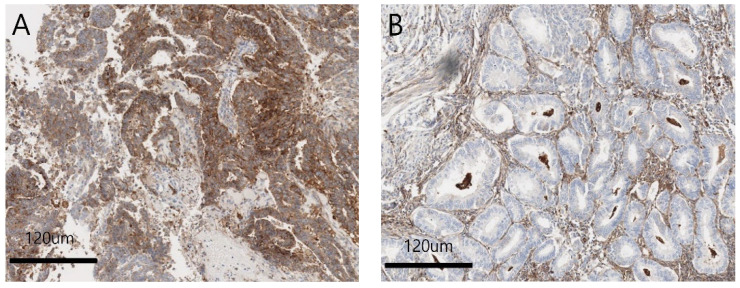
(**A**) Positive FcRn expression on endometroid endometrial tumor cells. Strong, diffuse cytoplasmic staining pattern is noted. (×100). (**B**) Negative FcRn expression on endometroid endometrial tumor cells. Cytoplasmic staining of FcRn is not shown. (×100).

The overall FcRn expression rate in patients with EC was 41.8% (17/41). FcRn-positive specimens showed a strong, diffuse cytoplasmic staining pattern (Figure 1A) compared to negative ones (Figure 1B), and cell staining was evenly distributed within the single core.

Table 2 shows the association of clinicopathological data in patients with EC with FcRn activity in cancer cell cytoplasm. The clinical feature with a statistically significant relationship to FcRn expression was FIGO stage. In endometrioid cancer cells of FIGO stage IA, FcRn was less frequently expressed than in other higher staged cases (*p* = 0.021). The relationship between tumor grade and FcRn expression showed a similar pattern to cancer stage, where low-grade cancer cells tended not to express FcRn compared to high-grade tumors, although it did not reach statistical significance (*p* = 0.064). Age, LVSI, and tumor size were not found to affect the expression of FcRn in EC.

### 3.3. FcRn Expression in Ishikawa Cell Culture

**Figure 2:**
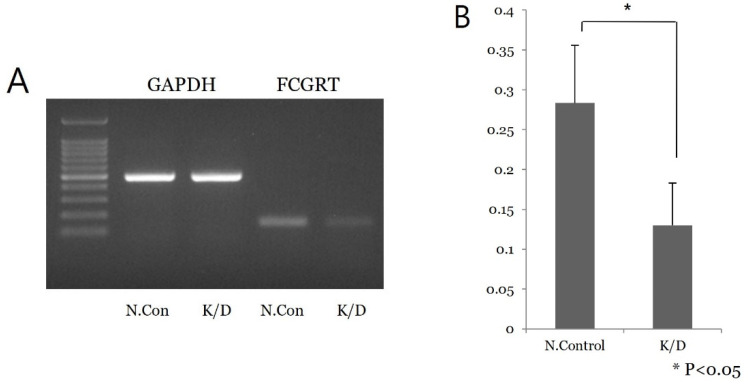
Semiquantitative PCR mRNA analysis in Ishikawa cell line. (**A**) A low signal of FCGRT mRNA of siRNA knock-down cell is noted. (**B**) Repeat test shows significant difference in mRNA expression between the control and knock-down groups (*p* < 0.05); N.con, scrambled RNA control; K/D, knock-down group by siRNA of FCGRT.

**Figure 3:**
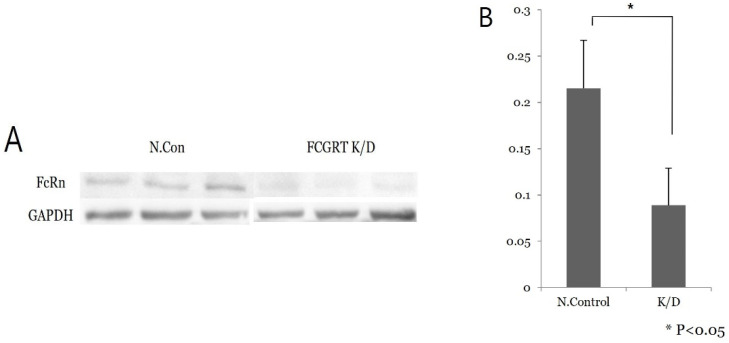
Western blot analysis. (**A**) Low signal of FcRn protein is noted in the knock-down group compared to the control group. (**B**) Repeat test shows significant difference in FcRn protein expression between the control and knock-down groups (*p* < 0.05); N.con, scrambled RNA control; K/D, knock-down group by siRNA of FCGRT.

**Figure 4:**
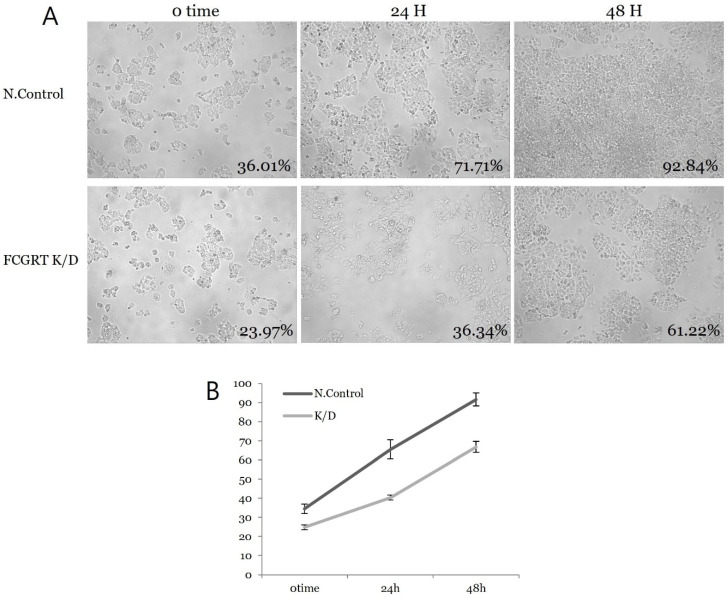
Cell proliferation analysis by confluence change. (**A**) Confluence change in each hotspot over time in both groups. (**B**) The slope of the plot shows no significant difference between the control and knock-down group; N.con, scrambled RNA control; K/D, knock-down group by siRNA of FCGRT.

siRNA knock-down experiments were conducted on the Ishikawa cell line three times, including PCR mRNA semiquantification, Western blot, and confluence change testing. In the semiquantitative mRNA experiment, FCGRT mRNA had a significant decrease in expression in the knock-down group compared to the control group (Figure 2). Western blot analysis confirmed a significant decrease in FcRn protein (Figure 3). Based on confluence change, the cell line culture function test showed that the knock-down group had a lower elevation in confluency over the initial 24 h. However, by the end, it exhibited almost identical changes in confluence compared to the control group (Figure 4). Confluence measurements for the knock-down group at three hotspots were 24.84% (SD 1.235) at 0 h, 40.35% (SD 1.235) at 24 h, and 66.80% (SD 2.849) at 48 h. In comparison, those of the control group were 34.45% (SD 2.494), 34.45% (SD 2.494), and 91.59% (SD 3.379), respectively.

## 4. Discussion

FcRn shares structural similarity with major histocompatibility complex class I molecules and functionally mediates the intracytoplasmic transfer of human serum albumin (HSA) and immunoglobulin G (IgG), both of which act as binding ligands to the Fc receptor [23]. Encoded by the FCGRT gene, FcRn exists as a heterochimeric receptor comprising three extracellular domains of heavy chains (α1, α2, and α3) and the β2-microglobulin light chain, forming a noncovalent connection with the transmembrane region [24,25]. Contrary to its name, FcRn is not limited to the neonatal period, but is known to be expressed throughout the entire human lifespan [26]. The peptide-binding groove of FcRn is occluded [23], granting it no peptide-binding properties beyond IgG and HSA [27]. FcRn functions in bidirectional transcytosis [20]. This transfer activity helps maintain the serum levels of IgG and HSA by shielding them from catabolism [28,29], a process known as recycling. The recycling activity prolongs the half-life of IgG and HSA, drawing scientific attention as a potential cancer therapeutic [30]. The dysregulation of cellular recycling of FcRn may explain the increased intracellular uptake of albumin, linking it to metabolic reprogramming, which supports FcRn’s involvement in tumor growth [31].

Not only in cancer therapeutics but also as a cancer prognostic factor, FcRn has been studied extensively. In 2022, Kim et al. reported that the negative expression of FcRn in non-small cell lung cancer cells is associated with significantly lower disease-free survival and decreased disease-specific survival in patients with TNM stage I disease [32]. Jensen et al. observed the downregulation of FCGRT, encoding FcRn, in progressive breast cancer patients [33]. The association between poor prognosis in hepatocellular carcinoma and non-small cell lung cancer and FCGRT downregulation has also been reported [19]. In our study, we revealed the expression of FcRn in endometrioid endometrial cancer, showing a correlation wherein EC cells with a stage higher than IA more frequently expressed FcRn (*p* = 0.021). Other clinicopathological features such as age, LVSI, myometrial invasion, and tumor size had no effect on FcRn expression in EC. In experiments on the Ishikawa cell line, the siRNA knock-down cell group exhibited a quantitatively lower expression of FCGRT mRNA and a lower signal of FcRn proteins compared to the scrambled RNA control group. Changes in confluence over time measured at three hotspots did not show a significant difference between groups.

In the non-small cell lung cancer study, it was elucidated that higher levels of FCGRT mRNA expression in both cancerous and noncancerous tissues are associated with a favorable prognosis [19]. Kim et al. also reported that FCGRT level may play a role in favorable outcomes in the lung cancer population [32]. However, in our study, we observed more frequent expression of FcRn in patients with high-staged EC. The interpretation of this result should be conducted with caution, as we did not evaluate the prognostic features of the disease.

EC presents robust evidence for molecular diagnosis, correlating well with treatment and prognosis. The recently revised FIGO stage guideline strongly advocates the performance of complete molecular classification, when feasible, for all ECs [7]. For prognostic risk group stratification, the molecular classification of EC cases is recommended. Integrating microscopic features with molecular characteristics is considered optimal to classify patients for prognostic purposes, particularly in regions with sufficient resources to adopt this technique [7]. Our patient was treated between 2002 and 2009, a period when molecular classification tests (POLE mut, MMRd, NSMP, and p53abn) were not frequently performed in Republic of Korea. Many advancements in the understanding of molecular factors in EC were not incorporated into the prognostic factor analysis of this study.

A growing body of evidence supports the role of FcRn in cancer therapeutics, particularly in albumin-based pharmacokinetics and antitumoral activity through antigen-presenting mechanisms [20]. Given the rapidly evolving nature of the field, it is reasonable to assert that research identifying the molecular characterization of EC, including the expression of FcRn and its clinical implications, holds a higher priority. Beyond studying FcRn expression in cancer cells concerning patient prognosis and cancer cell mechanisms, FcRn’s immune-related functions are being explored in obstetrics and gynecology. FcRn plays a crucial role in transferring maternal IgG antibodies to the fetus, a phenomenon observed during the COVID-19 pandemic [34]. A study by the John Hopkins group revealed that SARS-CoV-2 infection during pregnancy did not reduce FcRn expression in the placenta, but did decrease the maternal transfer of neutralizing antibodies [35]. In other words, FcRn, expressed by syncytiotrophoblasts in the placenta, is a protein critical for the mechanisms by which maternal antibodies are transferred to the fetus, playing a vital role in fetal health during maternal viral infections. The observed expression of FcRn in endometrioid carcinoma in this study suggests an essential role in the interaction between cancer cells and the human antibody immune system. While the association of human cancer cells with immunity has primarily focused on interactions with T cells and NK cells in cellular immunity, there are also reports of associations with antibody immunity. Mandal et al. reported that humoral immunity, especially IgA, is associated with tumor progression in EC, and may have therapeutic potential [36]. In the present study, we found that when the FIGO stage was higher than IA in endometrioid carcinoma, FcRn expression in cancer cells tended to increase. This may signify an interaction between human humoral immunity and cancer stage. Additionally, in cell line culture experiments, Ishikawa cell line proliferation did not change significantly in response to decreased FcRn expression, which is an important finding considering its association with human humoral immunity.

FcRn blocking treatment, utilizing the interaction mechanism between antibodies and FcRn, is gaining attention. MOISE Jr et al. reported that various autoimmune diseases in the perinatal period are caused by autoimmune antibodies, and suggested blocking FcRn to prevent antibody transfer for treating these conditions [37].

According to the results of the present study, FcRn blocking did not affect the rate of cell proliferation in vitro. However, in humans, where there are numerous antibodies against cancer cells, the situation is expected to be different. The role of FcRn in cancer cells remains unclear, but it is suspected to contribute to immune evasion mechanisms such as PD-1 and PD-L1 [38]. The function of FcRn in immunosurveillance for identifying tumor growth and bolstering antitumor immunity could be leveraged to specifically target immune cells expressing FcRn for more efficacious cancer immunotherapy [31]. To the best of our knowledge, our study is the first to evaluate the expression of FcRn in endometrioid carcinoma cells and provide the results of cell line experiments.

The limitations of our study include a relatively small number of cases, resulting in decreased statistical power in analyses. In addition, we only presented data from experiments using endometrioid endometrial cancer cells and Ishikawa cell culture. This hindered analyses of case-control comparative models, such as FcRn expression in EC versus non-malignant endometrium. As the mechanisms of action of FcRn in malignancies including EC are not understood, an experiment on normal endometrial cells for FcRn expression would have furthered our understanding of FcRn in the development of the EC cohort. Additionally, there is a lack of data regarding the prognostic features of the patients, and the findings of this study were not consistent with previous research on FcRn in cancer. In cancer-related studies, the impact of an intervention on disease-free survival or progression-free survival is important and provides important insights into cancer treatment and prevention. Unfortunately, it was impossible to obtain the molecular classification risks, as these were not available in our cohort, and we were also unable to address the impact of the expression of FcRn on the survival of the cohort. Lastly, an explanation for the underlying mechanism by which FcRn expression affects EC is lacking. Further studies are needed to elucidate FcRn expression in EC as a potential molecular marker. To our knowledge, our study is the first to evaluate the expression of endometrioid endometrial cancer cells. Endometrial cancer has robust evidence for molecular diagnosis, which correlates well with treatment and prognosis. FcRn expression levels in cancer have a higher potential for therapeutic exploitation. FcRn expression is known to play a pivotal role in the catabolism and anabolism of cancer growth, and the therapeutic approach of FcRn expression in malignancies is derived from its physiological action of molecular transport in cell membranes. Despite several limitations of our study, considering the rapidly evolving characteristic of the field it is reasonable to say that our study has value in that we showed the expression of FcRn in endometrioid EC and its relationship with clinicopathologic features of the cohort.

## 5. Conclusions

Our research reveals that 41.8% of women with EC exhibit FcRn expression. The FIGO stage was the clinical feature with a statistically significant association with FcRn expression. Other factors, such as age, LVSI, and tumor size, had no effect on FcRn expression in endometrial cancer. In FIGO stage IA EC cells, FcRn expression was less frequent than in other higher-stage cases. In Ishikawa cell line experiments, the siRNA knock-down cell group displayed quantitatively lower FCGRT mRNA expression and FcRn protein signal than the scrambled control group. The change in confluence over time, measured at three hot spots, did not show a significant difference between the groups. Further studies are needed to clarify the expression of FcRn in EC as a potential molecular marker and to better reflect the improved understanding of the complex nature of EC and its underlying biological behavior.

## Figures and Tables

**Table 1:** Clinicopathological features of 41 patients with endometrial cancer.

Variables		Value
Age, years (mean [range])		51 (35–78)
Menopause, n (%)	No	20 (48.8%)
	Yes	21 (51.2%)
FIGO stage, n (%)	IA	26 (63.4%)
	IB	8 (19.5%)
	IIA	2 (4.9%)
	3A	2 (4.9%)
	3C	3 (7.3%)
Tumor grade, n (%)	Low	38 (92.7%)
	High	3 (7.3%)
Tumor size, n (%)	<2 cm	8 (19.5%)
	2≥, <4 cm	13 (31.7%)
	≥4 cm	20 (48.8%)
LVSI, n (%)	Absent	36 (87.8%)
	Present	5 (12.2%)
Myometrial invasion, n (%)	<1/2	23 (56.1%)
	≥1/2	18 (43.9%)
FcRn expression, n (%)	Negative	24 (58.5%)
	Positive	17 (41.5%)

FIGO, International Federation of Gynecology and Obstetrics; LVSI, lymphovascular space invasion; FcRn, neonatal Fc receptor.

**Table 2:** Correlation between FcRn expression and clinicopathological features.

Variables	FcRn Expression		*p* Value
	Negative	Positive	
Age, years			0.105
≤60	22	12	
>60	2	5	
FIGO stage			0.021
IA	19	7	
Higher than IA	5	10	
Tumor grade			0.064
Low (G1,G2)	24	14	
High (G3)	0	3	
LVSI			0.084
Absent	23	13	
Present	1	4	

FcRn, neonatal Fc receptor; FIGO, International Federation of Gynecology and Obstetrics; LVSI, lymphovascular space invasion.

## Data Availability

The datasets used and/or analyzed during the current study are available from the corresponding author upon reasonable request.
